# Supplementation of *Lactobacillus curvatus* HY7601 and *Lactobacillus plantarum* KY1032 in Diet-Induced Obese Mice Is Associated with Gut Microbial Changes and Reduction in Obesity

**DOI:** 10.1371/journal.pone.0059470

**Published:** 2013-03-21

**Authors:** Do-Young Park, Young-Tae Ahn, Se-Hoon Park, Chul-Sung Huh, Sae-Rom Yoo, Rina Yu, Mi-Kyung Sung, Robin A. McGregor, Myung-Sook Choi

**Affiliations:** 1 Korea Yakult Co., Ltd., Yongin, Gyeonggi, Republic of Korea; 2 Center for Food and Nutritional Genomics, Kyungpook National University, Daegu, Republic of Korea; 3 Department of Food Science and Nutrition, Kyungpook National University, Buk-gu, Daegu, Republic of Korea; 4 Department of Food Science and Nutrition, University of Ulsan, Ulsan, Republic of Korea; 5 Department of Food and Nutrition, Sookmyung Women’s University, Seoul, Republic of Korea; Instutite of Agrochemistry and Food Technology, Spain

## Abstract

**Objective:**

To investigate the functional effects of probiotic treatment on the gut microbiota, as well as liver and adipose gene expression in diet-induced obese mice.

**Design:**

Male C57BL/6J mice were fed a high-fat diet (HFD) for 8 weeks to induce obesity, and then randomized to receive HFD+probiotic (*Lactobacillus curvatus* HY7601 and *Lactobacillus plantarum* KY1032, n = 9) or HFD+placebo (n = 9) for another 10 weeks. Normal diet (ND) fed mice (n = 9) served as non-obese controls.

**Results:**

Diet-induced obese mice treated with probiotics showed reduced body weight gain and fat accumulation as well as lowered plasma insulin, leptin, total-cholesterol and liver toxicity biomarkers. A total of 151,061 pyrosequencing reads for fecal microbiota were analyzed with a mean of 6,564, 5,274 and 4,464 reads for the ND, HFD+placebo and HFD+probiotic groups, respectively. Gut microbiota species were shared among the experimental groups despite the different diets and treatments. The diversity of the gut microbiota and its composition were significantly altered in the diet-induced obese mice and after probiotic treatment. We observed concurrent transcriptional changes in adipose tissue and the liver. In adipose tissue, pro-inflammatory genes (TNFα, IL6, IL1β and MCP1) were down-regulated in mice receiving probiotic treatment. In the liver, fatty acid oxidation-related genes (PGC1α, CPT1, CPT2 and ACOX1) were up-regulated in mice receiving probiotic treatment.

**Conclusions:**

The gut microbiota of diet-induced obese mice appears to be modulated in mice receiving probiotic treatment. Probiotic treatment might reduce diet-induced obesity and modulate genes associated with metabolism and inflammation in the liver and adipose tissue.

## Introduction

The gastrointestinal tract in an adult human contains approximately 10^12^ microorganisms per milliliter of luminal content and harbors approximately 500 to 1000 distinct bacteria species [1] collectively termed the microbiota. The gut microbiota plays an important role in the innate immune system and host metabolism[1]–[3]. There exists conflicting evidence whether the gut microbiota plays a role in obesity. Bäckhed et al. [4] observed that Germ-free (GF) B6 mice fed a chow diet appeared to be protected from excessive fat accumulation compared to conventionalized mice fed the same diet in both males and females. When GF animals were fed a Western-style, high-fat and sugar-rich diet, they appeared to be protected from diet-induced obesity [5]. However, another study reported that the absence of gut microbiota did not provide general protection from diet-induced obesity [6]. Mestdagh et al. [7] also reported that the total body fat content of GF C3H/Orl female mice fed a standard chow diet was not significantly different from that of conventional female mice fed the same diet. Therefore, the protection of GF mice from obesity appears to be dependent on diet and animal strain. Nevertheless, diet-induced obesity is reported to be associated with marked but reversible alterations in the mouse gut microbiota [8]. Hence the gut microbiota represents a therapeutic target with the potential to reverse existing obesity.

Probiotics consist of individual or multiple live bacteria species, which directly alter the gut microbiota, such as lactobacilli and bifidobacteria [9], [10]. Multiple *in-vivo* studies provide evidence that some probiotics can reduce diet-induced obesity in rodents[11]–[18], although there are reports of probiotics with no effect on body weight gain [19] or in some cases probiotics that actually cause weight-gain in rodents [20]. While many studies indicate probiotics intake causes functional changes, such as lower blood lipids in hyperlipidemic animals [21], evidence is lacking for the impact of probiotics containing individual or multiple bacterial species on gut microbiota diversity and composition of obese animals.

Importantly, different probiotic strains may have varying functional effects on the gut microbiota and obesity [22]. The apparent lack of microbiota changes in response to probiotics in some studies may be partly due to inter-individual variability in microbiota composition caused by genetic background, age, diet, or other environmental related factors. Some studies suggest a subset of microbial species appear to more widely spread colonizers of the human gastrointestinal tract, although no species appear to be universally present in all individuals [23], [24], while other studies suggest a common microbiome at the gene level may be shared between individuals [25], [26]. Pre-clinical models of diet-induced obesity in mice provide a useful way to assess physiologically relevant gut microbiota changes associated with both obesity and probiotic treatment, while controlling for the effects of genetic background, diet, age and other environmental factors on the gut microbiota.

The aim of this study was three fold as follows: to assess the functional effects of probiotic treatment on diet-induced obesity, to establish the effects of probiotic treatment on the gut microbiota of diet-induced obese mice and to assess the effects of probiotic treatment on the liver and adipose gene expression.

## Materials and Methods

### Animals, Diets and Experimental Design

Male C57BL/6J mice (n = 36) aged 4 weeks were purchased from Jackson Laboratories (Bar Harbor, USA). All mice were individually housed at a constant temperature and humidity (22±1°C, 55±10 percent) with a 12 h light/dark cycle. After 1 week allowing for adaptation, mice were fed a high-fat diet (n = 27, HFD; 20 percent fat and 1 percent cholesterol, w/w) to induce obesity or a normal diet (ND group, n = 9) for 8 weeks. Diet-induced obese mice were then randomly assigned to receive probiotics (HFD-probiotic group) or PBS (HFD-placebo group) for another 10 weeks, while the ND group was fed a normal diet. Yun et al. [27] tested the dose-dependent anti-diabetic effect of *Lactobacillus gasseri* (2×10^7^ ∼ 2×10^10^ cfu/day), and observed that diabetic *db*/*db* mice receiving the bacteria exhibited dose-dependent improvement for several metabolic biomarkers. In our recent study, to maximize the interventional effects of two probiotics, *L. curvatus* HY7601 and *L. plantarum* KY1032, we also used a high-dose of bacteria, 5×10^9^ cfu/day for each. *L. curvatus* HY7601 and *L. plantarum* KY1032 were isolated from Korean traditional fermented cabbage which is a rich source of *Lactobacillus* strains with potential probiotic properties [28]. *L. curvatus* HY7601 and *L. plantarum* KY1032 are reported to reduce adipogenesis in 3T3-L1 cells [29]. The probiotics were suspended in sterilized PBS and mixed with the diet immediately before being fed to the mice. Once the mice consumed the initial given amount of food, an additional amount of diet was added to the feed jar so that all the mice could consume all of the available food/probiotic mix every day. The composition of the diets was formulated based on the AIN-76 semi-synthetic diet (Table S1). A reference group (n = 9) was sacrificed to determine the accumulation of adipose tissue depots in diet induced obese mice after 8 weeks before probiotic treatment. Body weight was measured once a week. Before sacrifice, mice were fasted for 12 h and anesthetized with diethyl ether. Blood samples were taken from the inferior vena cava for plasma analysis. All adipose tissue depots were removed, rinsed with PBS and weighed. For real-time PCR analysis, the epididymal fat pad and liver tissue were frozen at −70°C right after removal. The experimental design was approved by the Ethics Committee at Korea Yakult Company Limited R&D center.

### Bacterial 16S rRNA Gene Amplification and Barcoded Pyrosequencing

For analysis of the microbial content, metagenomic DNA was extracted from the fecal samples of all mice using the QIAamp DNA stool mini kit (Qiagen, Netherlands) according to the manufacturer’s instruction. The extracted DNA was amplified using primers targeting the V1 to V3 hypervariable regions of the bacterial 16S rRNA gene (V1-9F: 5′-X-AC-GAGTTTGATCMTGGCTCAG-3′ and V3-541R: 5′-X-AC-WTTACCGCGGCTGCTGG-3′ where X denotes uniquely designed barcode for each mouse followed by a common linker AC). In this study, mixtures of barcodes with varied lengths (7 to 11 base pairs) were used. PCR reactions were carried out in a thermocycler (MJ Research, USA) under the following conditions: initial denaturation at 94°C for 5 min; followed by 20 cycles of denaturation at 94°C for 30 sec, annealing at 55°C for 45 sec, and elongation at 72°C for 1 min 30 sec. The amplified products were purified using resin columns, and 1 µg of PCR product for each mouse was mixed and subjected to pyrosequencing. The DNA sequencing was performed using the standard shotgun sequencing reagents and a 454 GS FLX Titanium Sequencing System (Roche, USA), according to the manufacturer’s instructions by Chunlab Inc., Republic of Korea. The length of the fragment of the 16S rDNA pyrosequenced ranged from 118 to 526 base pairs and the average length was 472 base pairs. The pyrosequencing data are available in the EMBL SRA database under the accession number ERP000935.

### Analysis of Bacterial 16S rRNA Gene Sequences

Pre-processing and taxonomic assignment of sequencing reads were conducted using a JAVA based bioinformatic pipeline (Chunlab Inc., Republic of Korea) described in a previous study [30]. First, sequencing reads from the different samples were separated by unique barcodes. Then, the barcode, linker, and PCR primer sequences at both sides were removed from the original sequencing reads. Individual collections of sequences were depleted of non-16S rRNA sequences and chimaeras using HMMER 3.0 (http://hmmer.janelia.org) and BLAST. Sequences shorter than 300 nt, having one or more ambiguous base calls, an average quality below 25, or showing no match with the 16S rRNA EzTaxon-e database (http://eztaxon-e.ezbiocloud.net/) [31] were also excluded from the subsequent analyses.

The trimmed sequences were assigned taxonomically via alignment with the EzTaxon-e database which includes not only species within the formal nomenclatural system, but also phylotypes that represent both cultured and uncultured entries in the GenBank public database [31]. Sequences with identity scores >97% were resolved at the species level, between 97% and 94% at the genus level, between 94% and 90% at the family level, between 90% and 85% at the order level, between 85% and 80% at the class level and between 80% and 75% at the phylum level based on EzTaxon-e [31]. If the similarity was below the cutoff value, the read was assigned to an “unclassified” group.

The beta diversity measure was calculated in order to compare between pairs of taxonomic communities using the MOTHUR software. All taxa found in one or both samples were placed on a phylogenetic tree. The UniFrac algorithm calculated the distance between two samples as the ratio of the sum of branch lengths leading to taxa from both samples to the sum of all branch lengths leading to all taxa [32]. The Unweighted Pair Group Method with Arithmetic Mean (UPGMA) [33] was used to cluster the pairwise distances of the samples, which indicates samples sharing community structures shown in a dendrogram. Phylogenetic distances between samples were also visualized with principal coordinate analysis (PCoA) as previously described [34].

For calculation of alpha diversity measures, sequences were clustered and assigned to operational taxonomic units (OTUs) using the CD-HIT algorithm [35]. The OTUs were inputted into the MOTHUR software [36] to generate diversity indexes such as rarefaction curves [37] and ACE richness estimator [38]. Rarefaction curves showed the OTUs observed as a function of the sampling effort, showing that a steep slope represents a large portion of the species diversity not yet sampled. The ACE richness estimator predicts the species richness based on the number of rarely occurring OTUs.

EzTaxon-e derived taxonomic communities were used to calculate the relative abundance (%) of bacteria at the phylum taxa level and species taxa level in each sample, as well as the core microbiota present in all samples. The present/absence of all microbial species at the species-level across samples was visualized in a heatmap generated with the R software. There is no universal consensus on a core microbiota definition, so we calculated the core microbiota shared by each group population at multiple thresholds between 50–100% of the mice in each experimental group. A Venn diagram was used to show core microbiota species shared by >78% of the mice in each experimental group regardless of treatment.

### Histological Analysis

Epididymal fat samples from each mouse were rinsed with sterilized PBS, fixed in 10 percent v/v formalin/PBS, and then embedded in paraffin for staining with hematoxylin and eosin (H&E). Images were obtained under a microscope (AxioObser Z1, Germany) at a magnification of ×200.

### Blood Analysis

Plasma leptin and insulin concentrations were determined using multiplex detection kits (Bio-Rad, USA) on the Bio-Plex Suspension array system (Bio-Rad, USA). The plasma total-cholesterol, triglyceride concentration, aspartate transaminase (AST) and alanine transaminase (ALT) activity were enzymatically determined using commercial kits (Asan Co., Republic of Korea). Plasma thiobarbituric acid-reacting substances (TBARS) were determined using a spectrophotometric method [31].

### RT-qPCR

Total RNA was extracted from adipose (100 mg) and liver (15 mg) tissues using an RNAqueous kit (Ambion, USA). Total RNA (2 µg) was reverse-transcribed into cDNA with the high-capacity RNA-to-cDNA kit (Applied Biosystems Inc., USA). The cDNA was amplified on a 7500 Real Time PCR System (Applied Biosystems Inc., USA) using mouse-specific Taqman probe sets (Table S2) and normalized to GAPDH. The data are presented as the means ± SE.

### Statistical Analysis

All data were presented as the means ± SE. For metabolic and gene expression data analysis, significant differences between groups (HFD+placebo versus ND, HFD+probiotic versus HFD+placebo) were determined using unpaired Student’s t-test. For the relative abundance analysis of the gut microbiota, significant differences between groups (HFD+placebo versus ND, HFD+probiotic versus HFD+placebo) were determined using Kruskal–Wallis one-way analysis of variance with Bonferroni correction to control for multiple comparisons. All values were considered statistically significant when p<0.05.

## Results

### Effects of Probiotic Treatment on Body Weight, Fat Mass and Adipocytes of Diet-induced Obese Mice

**Figure 1:**
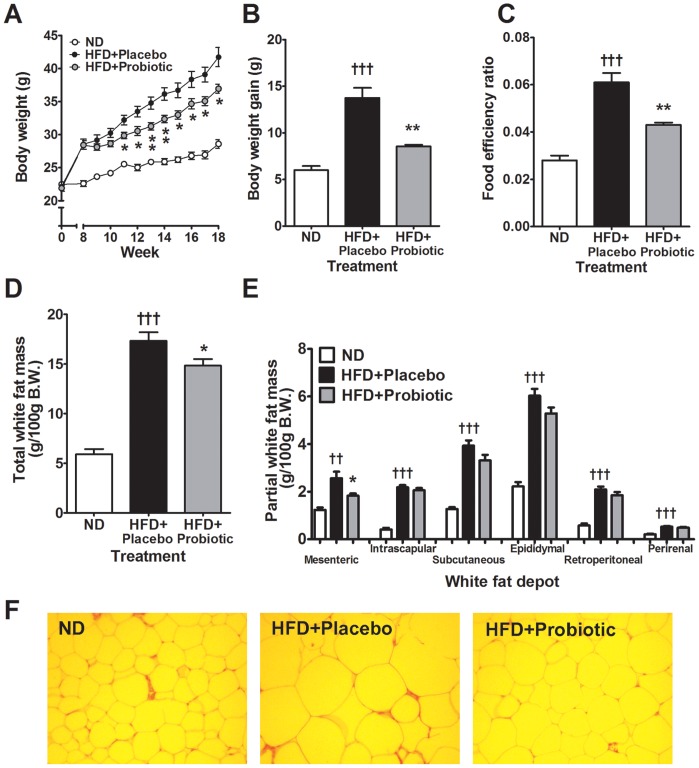
High-fat diet and probiotic effect on body weight, fat mass and adipocyte morphology. Effects of probiotic treatment on (A) body weight, (B) body weight gain, (C) food efficiency ratio, (D) total white fat mass, (E) partial white fat mass and (F) epididymal adipocyte morphology. Results are expressed as the means ± SE. Significant differences HFD-placebo versus ND are indicated as ^††^p<0.01 ^†††^p<0.001. Significant differences between HFD-probiotic versus HFD-placebo are indicated as *p<0.05, **p<0.01. Epididymal adipose tissue morphology shown at ×200 magnification.

Diet-induced obesity was induced for over 8 weeks (28.55±0.77 g versus 22.59±0.45 g, HFD+placebo and ND group respectively) (Figure 1A), but following probiotic treatment, HFD-induced body weight gain was 38 percent lower (p<0.01) in the probiotic group (8.53±0.20 g) than in the HFD+placebo group (13.75±1.07 g) (Figure 1B). Hence, after 10 weeks, the average body weight was 11 percent lower (p<0.05) in the HFD+probiotic group compared to the HFD+placebo group, although HFD+probiotic mice still gained more weight than those mice consuming the ND (Figure 1A).

Food intake in the probiotic group was not significantly different compared to the HFD+placebo mice, although mice receiving probiotics consumed 7∼8% less food overall (Figure S1A). The food efficiency ratio (FER) represents total grams of body weight gained on a test food divided by the total grams of food consumed during an animal feeding study. In the HFD mice the FER was significantly increased compared to the ND-fed mice, reflecting greater efficiency of HFD on weight gain, conversely the FER was reduced by 29 percent (p<0.01) in the probiotic treatment group (Figure 1C) reflecting lower weight gain per grams of food consumed.

Liver toxicity biomarkers alanine transaminase (ALT) and aspartate transaminase (AST) were significantly elevated in the HFD compared to ND mice (Figure S1B and S1C). Conversely, plasma ALT was reduced by 70 percent (p<0.05) in the HFD+probiotic group, and plasma AST was non-significantly reduced by 20 percent compared to the HFD+placebo group.

White fat mass was significantly higher in the HFD+placebo group compared to the ND group after 18 weeks (p<0.001, Figure 1D). Fat accumulation was lower in the mice that received probiotic treatment since total white fat mass gain was 31 percent lower in the HFD+probiotic group (∼5.69 g) than in the HFD+placebo group (∼8.19 g). Hence, total white fat mass was reduced by 14 percent in the HFD+probiotic compared to the HFD+placebo group (p<0.05, Figure 1D). Analysis of adipose tissue depots revealed consistently higher mesenteric, intrascapular, subcutaneous, epididymal, retroperitoneal and perirenal fat in the HFD+placebo mice compared to the ND-fed mice. The mesenteric adipose tissue depot was reduced by 28 percent (p<0.05) in the HFD+probiotic mice compared to the HFD+placebo mice (Figure 1E). Furthermore, histological analysis of white (epididymal) adipose tissue showed the size of the adipocytes was markedly increased in the HFD+placebo mice versus the ND mice but reduced in the mice receiving probiotic treatment (Figure 1F).

### Effects of Probiotic Treatment on the Indexes of Gut Microbial Diversity in Diet-induced Obese Mice

To establish the gut microbiota changes caused by diet-induced obesity and the effect of probiotic treatment on the gut microbiota we performed fecal pyrosequencing. A total of 151,061 pyrosequencing reads for the fecal microbiota were analyzed with a mean of 6,564, 5,274 and 4,464 reads for the ND, HFD+placebo and HFD+probiotic groups, respectively (Table S3). The full pyrosequencing dataset is available in the EMBL SRA database, accession ERP000935. Beta diversity analysis using the hierarchical clustering algorithm UPGMA (Unweighted Pair-Group Method with Arithmetic mean) and dimensionality reduction using PCoA (Principal Coordinates Analysis) showed that the fecal samples were intermingled and did not form distinct non-overlapping clusters (Figure S2A and S2B).

**Figure 2:**
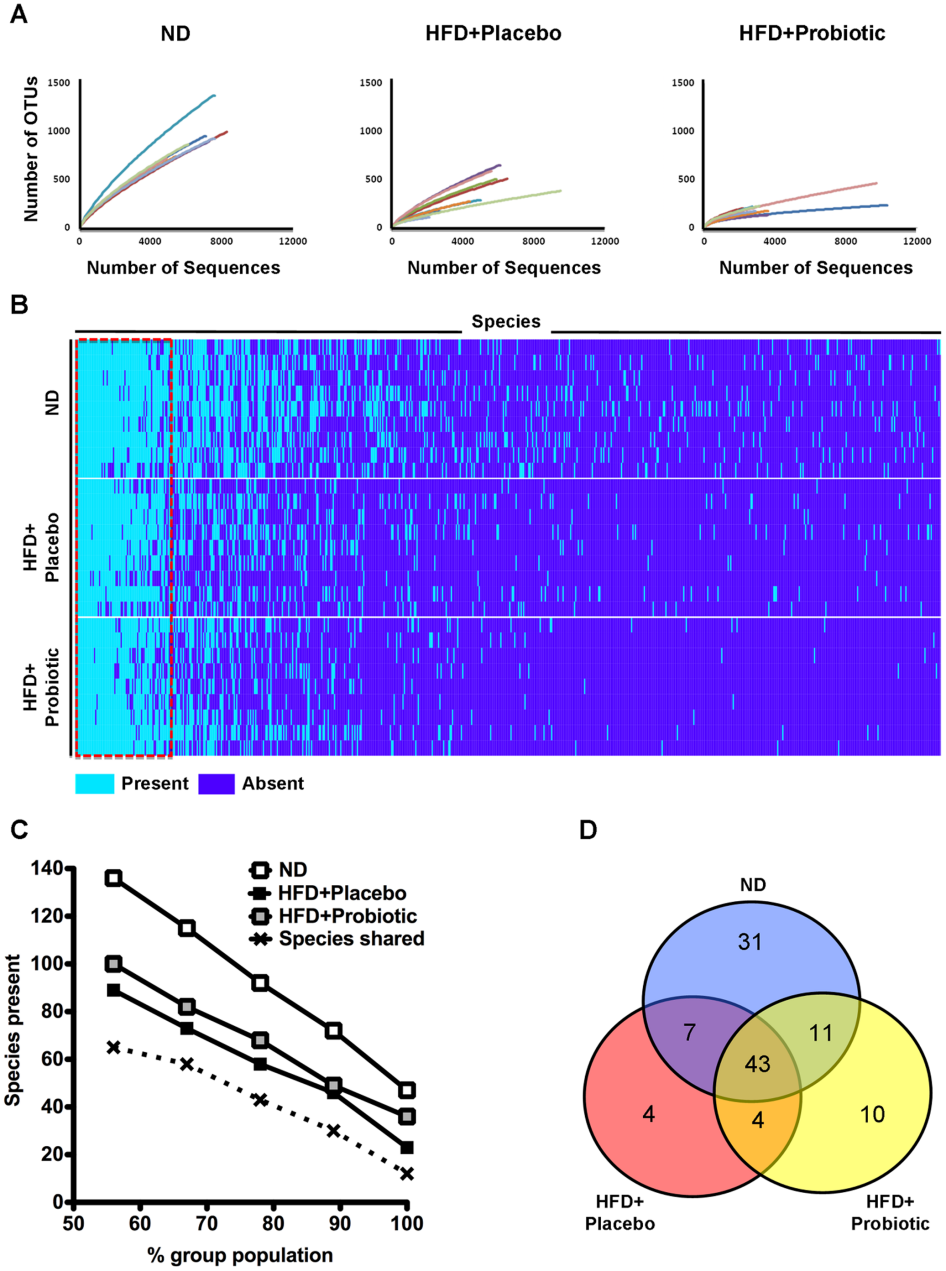
Shared microbiota and species diversity. (A) Rarefaction analysis of 151,061 pyrosequencing reads of 16S rRNA from feces using OTUs at a 97 percent sequence similarity cut-off value. (B) Microbiota species present or absent in ND, HFD+placebo and HFD+probiotic mice. Dark blue indicates species absent in the gut microbiota, light blue indicates species present in the gut microbiota. (C) Shared microbiota within and between groups varies depending on the percent group population cut-off used to define the shared microbiota. (D) Venn diagram of overlap in species shared by >78 percent of the mice from each group.

To assess alpha diversity, pyrosequencing reads were assigned into operational taxonomic units (OTUs) with 97 percent sequence similarity and represented using rarefaction curves. Rarefaction analyses indicated individual rarefaction curves of the probiotic group appeared to plateau in the majority of samples (Figure 2A and S3), in agreement with the ACE richness estimator (Table S3). The number of OTUs observed and estimated showed that the diversity of gut microbiota of the HFD+placebo mice was lower (p<0.001) than that of the ND mice (Figure 2A, Table S3). The gut microbiota diversity was further lowered (p<0.05) in the HFD+probiotic compared to the HFD+placebo mice (Figure 2A, Table S3).

### Gut Microbiota Shared between Mice Independent of Diet and Treatment

There is debate regarding whether a shared microbiota community exists between mammals, which is an important assumption in pre-clinical studies comparing gut microbiota between treatment groups; therefore, we determined the presence or absence of species across all samples and how many species were shared between samples. As shown in Figure 2B, there were 682 species detected overall (563, 355 and 265 species in the ND, HFD and HFD-probiotic group respectively), of which about a tenth of the microbial species (in the dotted red box) were present in over half of all the samples regardless of the treatment. Specifically, the number of bacterial species shared between the groups is dependent on how many mice in each group were used for the comparison between the groups (Figure 2C). For instance, when comparing 100% (9/9), 89% (8/9), 78% (7/9), 67% (6/9) and 56% (5/9) of the population in each group with one another, the number of species shared among all groups was 12, 30, 43, 58 and 65, respectively (Table S4). Figure 2D shows the number of shared species when the 78% criterion was applied; 43 species were shared among all three groups and many more species were shared between two groups or within each group.

### Effects of Probiotic Treatment on the Gut Microbiota Composition of Diet-induced Obese Mice

**Figure 3:**
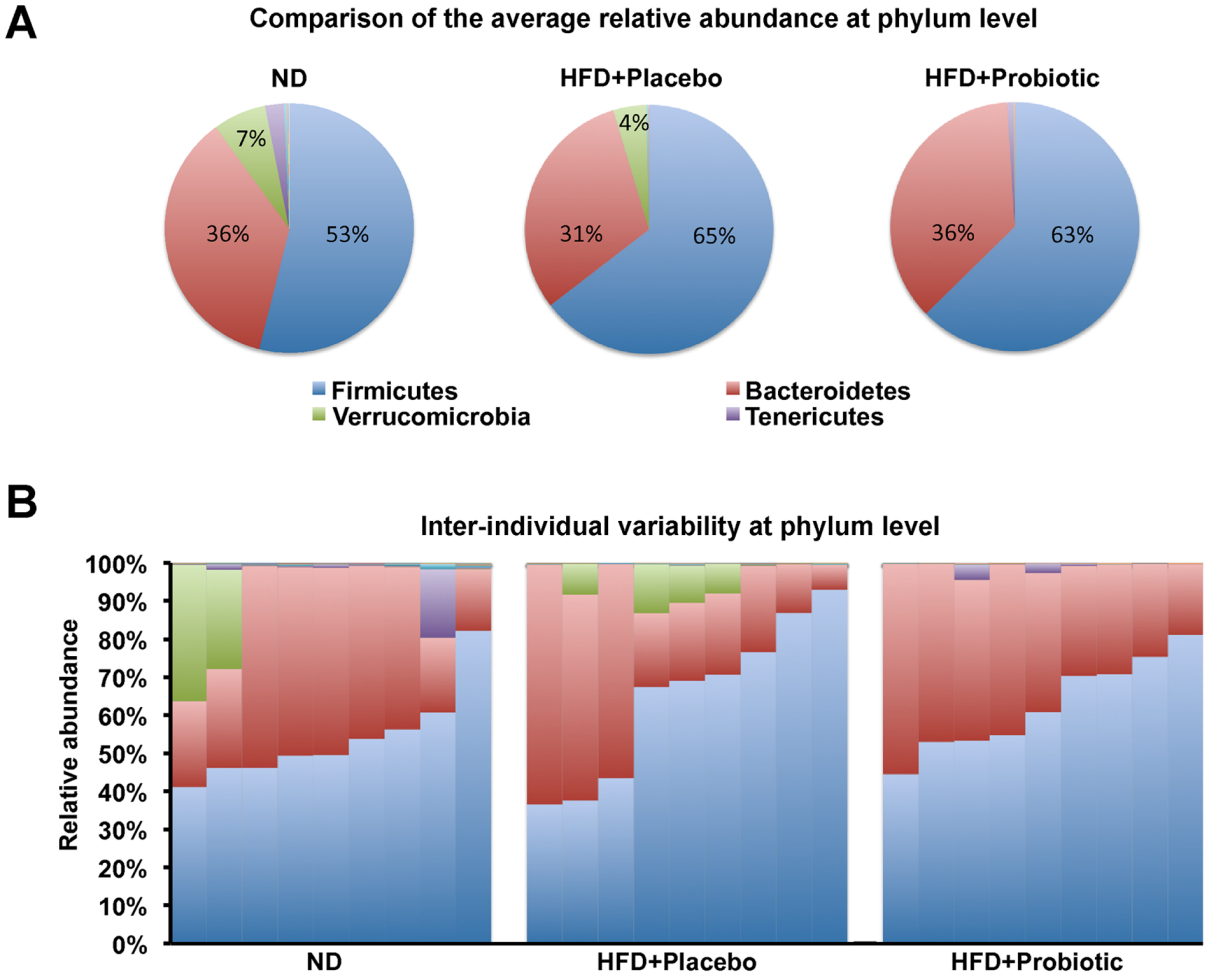
High-fat diet and probiotic effect on the gut microbiota composition at the phylum level. (A) The average relative abundance of the major microbial phyla in ND, HFD+placebo and HFD+probiotic mice. (B) Inter-individual variability in the relative abundance of the major microbial phyla in ND, HFD+placebo and HFD+probiotic mice. Values presented are percentage of relative abundance with respect to total bacterial sequences.

Gut microbial compositions for the most abundant two phyla *Firmicutes* and *Bacteroidetes* were not significantly different between the groups (Figure 3A, Table S5). Among the other phyla, the relative abundance of phylum *Tenericutes* was significantly lower in the HFD+placebo group compared to the ND group. In addition, the phylum *Verrucomicrobia* was absent and *Proteobacteria* was significantly lower in the probiotic treated group compared to the placebo treated HFD fed mice (Table S5). Inter-individual variability was also apparent at the phylum level within all groups (Figure 3B).

**Figure 4:**
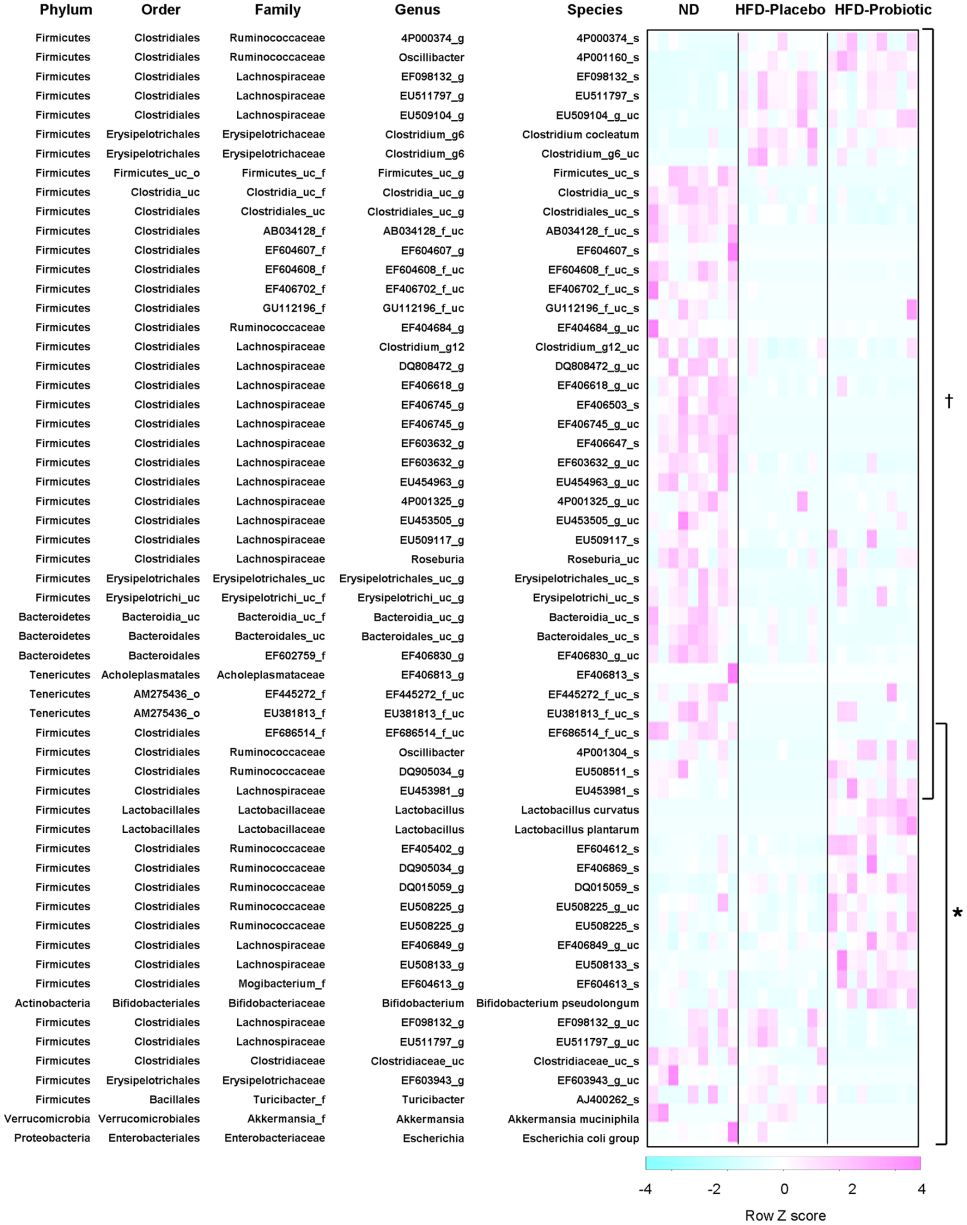
High-fat diet and probiotic effect on gut microbiota composition at the species level. Heatmap of the relative abundance of microbial species altered by diet-induced obesity or probiotic treatment. Data represents row scaled Z-scores. Significant differences between HFD-placebo versus ND are indicated as ^†^p<0.05. Significant differences between HFD+probiotic versus HFD+placebo are indicated as *p<0.05.

Next, we analyzed the differences in the gut microbiota composition at the species level. The relative abundance was determined for 682 gut bacterial species detectable in this study. We found that the relative abundance of 40 species was significantly (p<0.05) different in mice with diet-induced obesity, with an increase of 7 species (Figure 4, Table S6) and a decrease of 33 species (Figure 4, Table S7). The relative abundance of 4 species (EF686514_f_uc_s, 4P001304_s, EU508511_s and EU453981_s) belonging to the EF686514_f, *Ruminococcaceae* and *Lachnospiraceae* families of the order *Clostridiales* and phylum *Firmicutes*, which were decreased by HFD, were increased in mice receiving probiotic treatment (Figure 4, Table S7).

Gut microbial species not associated with changes caused by diet-induced obesity also appeared to be affected in mice receiving probiotic treatment. The relative abundance of 18 more species from diverse taxonomic orders including *Lactobacillales*, *Clostridiales*, *Bifidobacteriales*, *Erysipelotrichales*, *Bacillales*, *Verrucomicrobiales*, *Enterobacteriales* appeared to be different in the probiotic treated mice, with an increase of 11 species (Figure 4, Table S8) and a decrease of 7 species (Figure 4, Table S9). Following probiotic treatment *L. curvatus* and *L. plantarum* species were present at a relative abundance of 0.362±0.070 percent and 0.065±0.021 percent for all bacterial sequences in the HFD-probiotic group (Table S8). Furthermore, the relative abundance of endogenous *Bifidobacterium pseudolongum* was higher (p<0.01) in the HFD+probiotic mice compared to the HFD+placebo mice, although *B. pseudolongum* was not externally administered (Figure 4, Table S8).

### Effects of Probiotic Treatment on Blood Lipid and Hormone Levels in Diet-induced Obese Mice

**Figure 5:**
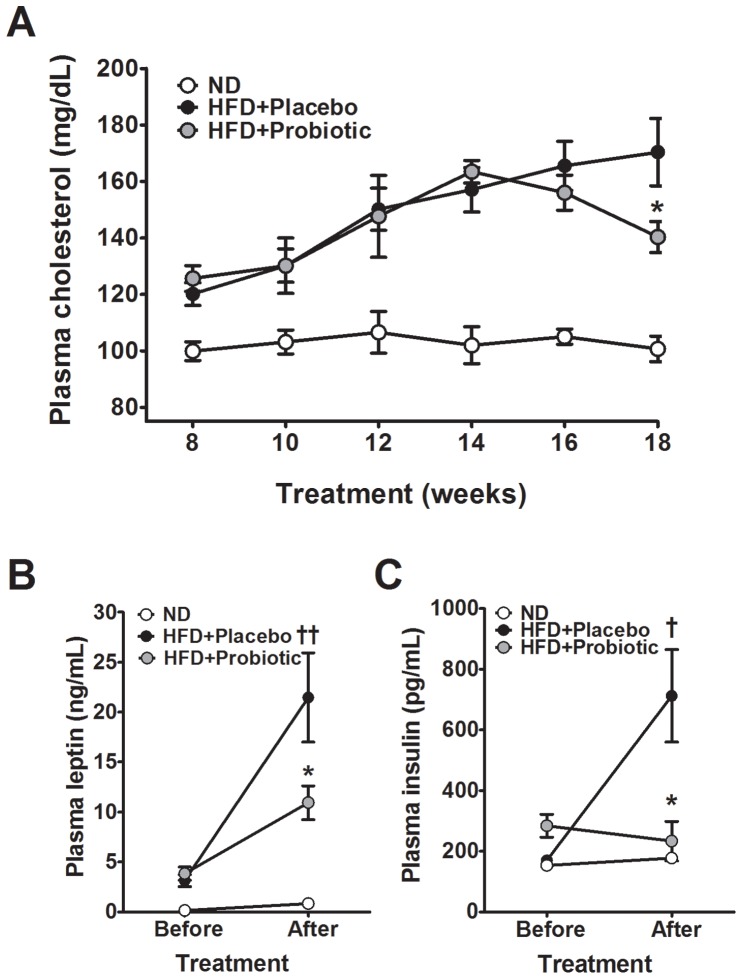
High-fat diet and probiotic effect on plasma cholesterol, leptin and insulin. Effects of probiotic treatment on (A) plasma cholesterol, (B) leptin and (C) insulin in diet-induced obese mice. Results are expressed as the means ± SE. Significant differences between HFD-placebo versus ND are indicated as ^†^p<0.05, ^††^p<0.01. Significant differences between probiotic versus HFD are indicated as *p<0.05.

We established a time-course of changes in plasma cholesterol and triglycerides in our pre-clinical model of diet-induced obesity. Cholesterol was consistently elevated in the HFD fed mice compared to the ND fed mice (Figure 5A), but plasma triglycerides were not significantly different (data not shown). Importantly, the time-course analysis revealed plasma cholesterol was significantly reduced by 17 percent (Figure 5A, p<0.05) after 10 weeks of treatment in the HFD+probiotic mice compared to the HFD+placebo mice.

Plasma leptin and insulin were both elevated in our experimental model of diet-induced obesity. Conversely, plasma leptin and insulin were lowered by 49 percent (p = 0.048) and 67 percent (p = 0.025), respectively, in the HFD+probiotic mice compared to the HFD+placebo mice (Figure 5B and 5C). Plasma TBARS, a measure of oxidative stress, was significantly elevated in the HFD mice compared to the ND mice (p<0.05); however, probiotic treatment caused a 30 percent decrease in TBARS compared to the placebo, but the difference was not significant (data not shown).

### Effects of Probiotic Treatment on Gene Expression in the Adipose Tissue of Diet-induced Obese Mice

**Figure 6:**
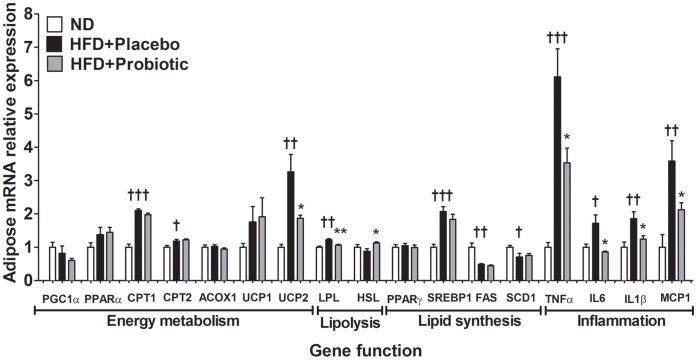
High-fat diet and probiotic effect on gene expression in epididymal fat. Results are expressed as the means ± SE. Significant differences between HFD-placebo versus ND are indicated as ^†^p<0.05, ^††^p<0.01, ^†††^p<0.001. Significant differences between HFD+probiotic versus HFD+placebo are indicated as *p<0.05, **p<0.01, ***p<0.001.

Next, we measured gene expression in epididymal adipose tissue. HFD intake significantly increased the expression of epididymal adipose tissue genes controlling inflammation (TNFα, IL6, IL1β, MCP1) and fatty acid oxidation (CPT1, CPT2), as well as UCP2, LPL and SREBP1 (Figure 6). Conversely, HFD intake significantly decreased the expression of FAS and SCD1 genes. We found that TNFα (−42 percent), IL6 (−50 percent), IL1β (−33 percent), MCP1 (−41 percent), UCP2 (−43 percent) and LPL (−13 percent) gene expression in the epididymal fat were significantly lower in the HFD+probiotic mice compared to the HFD+placebo mice (Figure 6). Conversely, epididymal HSL (+28 percent) gene expression was significantly higher in the HFD+probiotic mice compared to the HFD+placebo mice.

### Effects of Probiotic Treatment on Lipid and Cholesterol Metabolism Gene Expression in the Liver of Diet-induced Obese Mice

**Figure 7:**
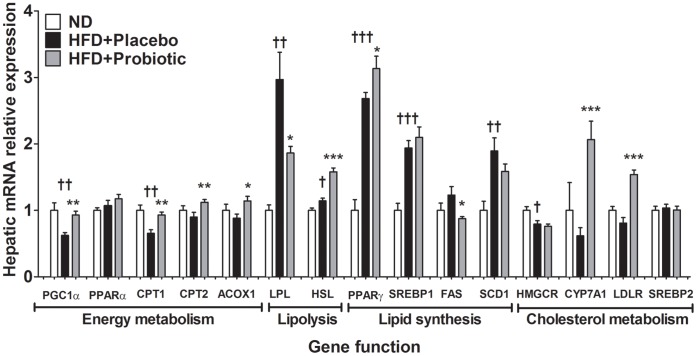
High-fat diet and probiotic effect on gene expression in liver. Results are expressed as the means ± SE. Significant differences between HFD-placebo versus ND are indicated as ^†^p<0.05, ^††^p<0.01, ^†††^p<0.001. Significant differences between HFD+probiotic versus HFD+placebo are indicated as *p<0.05, **p<0.01, ***p<0.001.

We also examined the mRNA levels of genes related to lipid metabolism in the liver. As shown in Figure 7, HFD intake significantly decreased the expression of genes involved in fatty acid oxidation (PGC1α, CPT1) and increased the expression of genes involved in the regulation of adipogenesis (PPARγ, SREBP1, SCD1) and lipogenesis (LPL). Conversely, fatty acid oxidation related gene expression including PGC1α (+49 percent), CPT1 (+42 percent), CPT2 (+25 percent) and ACOX1 (+30 percent) was significantly higher in the HFD+probiotic mice compared to the HFD+placebo mice. In addition, LPL (−37 percent) and FAS (−41 percent) gene expression was significantly lower in the HFD+probiotic mice compared to the HFD+placebo mice. Additionally, the HSL mRNA level was significantly higher in the HFD mice compared to the ND mice and increased another 38 percent in mice receiving probiotic treatment (p<0.001). Finally, CYP7A1 and LDLR gene mRNA levels were significantly higher by 235 percent and 90 percent, respectively, in the probiotic treated mice compared to the placebo treated mice.

## Discussion

### Probiotic Use is Correlated with a Reduction in Diet-induced Obesity

The relationship between food intake and weight gain based on a given diet is very important in nutrition intervention studies. For this reason, the food efficiency ratio (FER) was used as an index for the efficiency of the given food. While mice treated with *L. curvatus* HY7601 and *L. plantarum* KY1032 showed 7∼8% (∼0.2 g) less food intake compared to the placebo-treated mice, probiotics-treated mice showed a greater reduction in body weight gain (∼35% reduction) and total white fat mass gain (∼31%) compared to the placebo-treated mice. These results indicate that the probiotics added diet does play a role in suppressing body weight gain compared to its control diet. Of note, fat accumulation was predominantly lower in the mesenteric adipose depot. There is some evidence to indicate other probiotics such as *L. gasseri* SBT2055 also reduce adipocyte size in the mesenteric fat depot of rats [39], which may be partly attributable to its close proximity to the gastrointestinal tract. A longer probiotic treatment duration may lead to greater changes in all visceral fat depots.

### Probiotics Appear to Modulate the Gut Microbiota of Diet-induced Obese Mice

Probiotics may provide a way to alter the gut microbiota naturally and they partly explain the reduction in fat accumulation in response to probiotic interventions. However, there was a recent report that *L. acidophilus* NCDC 13 supplementation has no detectable effect on obese animals [19]. Some of the discrepancies between findings may be due to the strain-specific effects of probiotics. Another contributing factor is inter-individual variability in the gut microbiota between individuals, which may be attributable to differences in genetic background, age and diet [40]. Therefore, it is essential when assessing the effect of probiotics on obesity and the gut microbiota that confounding factors such as genetic background, age, sex and diet are controlled. To minimize inter-individual variability in the gut microbiota, we used mice with the same genetic background, age and sex, and observed that about a tenth of all detected microbial species were shared by over half of the mice regardless of diet or treatment, which is an essential assumption for preclinical studies of probiotic interventions.

We tried to ensure the reliability of the species identity determined by pyrosequencing. We sequenced the 16 s rRNA gene region of *L. curvatus* HY7601 using the 16 s rRNA universal primers (27F, 1492R) and compared it to *L. curvatus* HY7601 pyrosequences. Both sequences were perfectly aligned. *L. plantarum* KY1032 also showed alignment between both sequences proving the reliability of the species identity carried out by pyrosequencing, in at least two species (Table S10).

At the phylum level, there is some evidence which indicates the ratio of *Bacteroidetes*:*Firmicutes* is decreased in diet-induced obese mice, *ob/ob* mice [41], obese humans [42], and conversely increased by weight-loss [42]. In contrast, other studies, particularly in humans, suggest the *Bacteroidetes*:*Firmicutes* ratio is not a factor in human obesity and the *Bacteroidetes*:*Firmicutes* ratio appears to be unrelated to diet [43]–[45]. In the present study, we did not find significant differences in the relative abundance of *Bacteroidetes* and *Firmicutes* among the experimental groups.

At the species level, we found significant differences between the groups. The relative abundance of 40 species was altered by diet-induced obesity, 4 of which were altered in mice receiving probiotic treatment. In addition, 18 species were different in mice with probiotic treatment independently of diet-induced obesity. Remarkably, the relative abundance of *Bifidobacterium pseudolongum* was about 10 times higher in the HFD-probiotic group than in the HFD-placebo group in present study, which appears to be correlated with the suppression of body weight gain or body fat reduction in diet-induced obese mice. Recent reports suggest that certain members of the genus *Bifidobacterium* have conferred health-promoting or probiotic effects [46], [47].

Alongside modulation of the gut microbiota at the species level, we found that mice in the probiotic treatment group also had lower gut microbiota diversity. Low gut microbiota diversity is usually a hallmark of intestinal dysbiosis. Our findings of reduced microbial diversity alongside reduced weight-gain are consistent with evidence from obese mice treated with antibiotics [48], [49] or wheat arabinoxylan [50]. In addition, germ-free mice which lack gut microbiota appear to be protected against diet-induced obesity [5], [7]. Some probiotic strains may reduce the diversity of the gut microbiota by either increased competition for nutrients or alternatively by production of antimicrobial peptides that reduce microbial growth [10]. Further study is required to establish whether probiotic strains commonly reduce the diversity of the gut microbiota and whether there are any long-term consequences of prolonged probiotic supplementation.

### Inflammation and Lipid Metabolism Related Gene Expression in Adipose and Liver Tissue Appears to be Altered by Probiotics

Chronic low-grade inflammation is a characteristic of obesity [51]. Inflammatory cytokine related genes including TNFα, IL6, IL1β and MCP1, which were increased in parallel with plasma insulin levels in the diet-induced obese mice, were reduced in mice receiving probiotic treatment. Several potential mechanisms may explain the reduction of pro-inflammatory cytokine expression induced in mice receiving probiotic treatment. Pathogenic gut microbials are reported to stimulate LPS production and secretion from intestinal epithelial cells, which can then bind cytokine receptors on hepatocytes and adipocytes triggering pro-inflammatory cytokine release [52]. Intestinal barrier function is reported to be improved by some probiotic strains [53], hence reducing LPS release from the intestinal epithelial cells, leading to decreased pro-inflammatory cytokine production in adipose tissue. It will be important in future studies to assess whether the probiotic strain we used herein leads to reduced inflammatory-related gene expression due to alteration in the intestinal barrier function or due to some other factors.

Energy metabolism related gene expression was increased in the liver of probiotic treated mice, including PGC1α, CPT1, CPT2 and ACOX1 which suggests some of the differences in fat accumulation may have been due to increased mitochondrial oxidation of long chain fatty acids [54] and increased fatty acid oxidation in the liver [55]. Although further studies are needed to determine whether probiotics exert any direct effect on energy metabolism genes or whether probiotic effects are predominantly mediated by changes in the gut microbiota.

Hepatic cholesterol uptake and bile synthesis related genes including LDLR and CYP7A1, were also higher in the probiotic treated mice, which was consistent with the lower cholesterol levels in the probiotic treated mice. Probiotic effects on cholesterol levels have been primarily attributable to lowered gastrointestinal cholesterol absorption or increased cholesterol excretion, mediated via bile metabolism [56]. In both adipose and liver tissues, lower LPL gene expression and higher HSL gene expression in the probiotic treated mice suggests that probiotic treatment may reduce fatty acid uptake and augment lipolysis [57], [58]. We acknowledge that the effects of the probiotic treatment on a relatively small panel of genes related to inflammation and metabolism were assessed. In future studies, it may be worthwhile to conduct global gene and proteomic profiling to further elucidate the underlying response of metabolic tissues to probiotic treatment.

### Conclusions

Body weight gain was reduced in diet-induced obese mice treated with *L. curvatus* HY7601 and *L. plantarum* KY1032. The gut microbiota was also different in diet-induced obese mice receiving probiotic treatment. Furthermore, energy metabolism and inflammation related genes in liver and adipose tissue were also concomitantly different in mice receiving probiotic treatment. Taken together, these findings suggest *L. curvatus* HY7601 and *L. plantarum* KY1032 supplementation might modulate the gut microbiota, at least in mice, and may provide a natural alternative to combat obesity. However, full scale trials in humans are required.

## Supporting Information

Figure S1 **High-fat diet and probiotic effect on food intake and toxicity biomarkers.** Effects of probiotic treatment on (A) food intake, (B) plasma ALT(Alanine transaminase) toxicity and (C) AST(Asparatate transaminase) toxicity in diet-induced obese mice. Results are expressed as mean ± SE. Significant differences between HFD-placebo versus ND are indicated as ^†^p<0.05. Significant differences between HFD+probiotic versus HFD+placebo are indicated as *p<0.05.
(TIF)

Figure S2 **Clustering of samples based on gut microbiota communities.** (A) UPGMA (Unweighted Pair-Group Method with Arithmetic mean) of samples from ND, HFD+placebo and HFD+probiotic group. (B) PCoA (Principal Coordinates Analysis) of samples from ND, HFD+placebo and HFD+probiotic group.
(TIF)

Figure S3 **Rarefaction curves including 95% confidence intervals.** Rarefaction analysis of 151,061 pyrosequencing reads of 16S rRNA from faeces at a 97 percent sequence similarity cut-off value. Rarefaction curves including 95% confidence intervals were constructed using MOTHUR software.
(TIF)

Table S1 **Composition of experimental diet.**
(DOC)

Table S2 **Catalog numbers of Taqman probes.**
(DOC)

Table S3 **The number of sequences analyzed, observed OTUs and estimated OTUs.**
(DOC)

Table S4 **The total detectable species and core microbiota shared between groups.**
(XLSX)

Table S5 **The relative abundance at the phylum level.**
(DOC)

Table S6 **Microbial species elevated by diet-induced obesity.**
(DOC)

Table S7 **Microbial species reduced by diet-induced obesity.**
(DOC)

Table S8 **Microbial species elevated in mice receiving probiotic treatment.**
(DOC)

Table S9 **Microbial species reduced in mice receiving probiotic treatment.**
(DOC)

Table S10 **Comparison between 16 s RNA gene sequenced using universal primers and pyrosequencing primers.**
(DOCX)
