# Supplementary figures and images for: Supplementation of Lactobacillus curvatus HY7601 and Lactobacillus plantarum KY1032 in Diet-Induced Obese Mice Is Associated with Gut Microbial Changes and Reduction in Obesity
Source: PLoS One. 2013 Mar 21;8(3):e59470. doi: 10.1371/journal.pone.0059470 (PMC3605452; doi:10.1371/journal.pone.0059470)

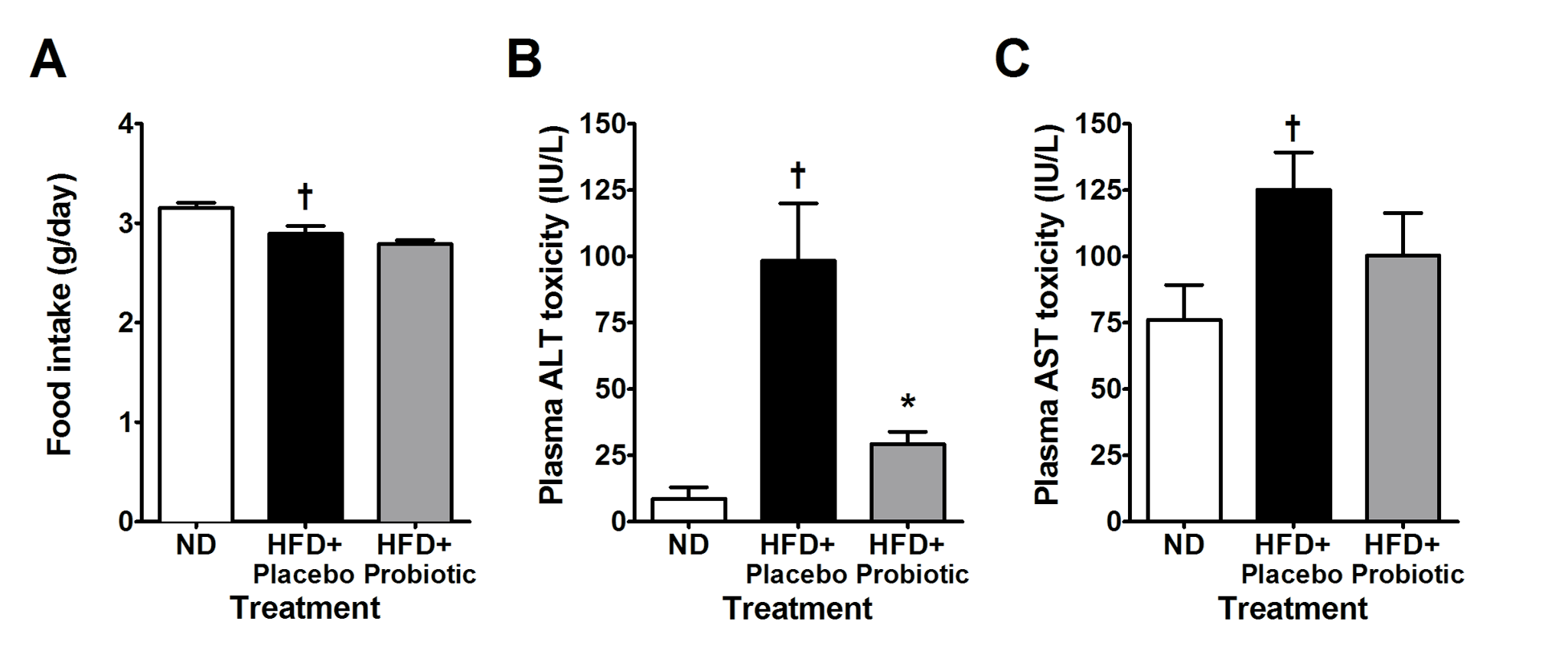

Supplement: Figure S1 — High-fat diet and probiotic effect on food intake and toxicity biomarkers. Effects of probiotic treatment on (A) food intake, (B) plasma ALT(Alanine transaminase) toxicity and (C) AST(Asparatate transaminase) toxicity in diet-induced obese mice. Results are expressed as mean ± SE. Significant differences between HFD-placebo versus ND are indicated as †p<0.05. Significant differences between HFD+probiotic versus HFD+placebo are indicated as *p<0.05. (TIF) [file pone.0059470.s001.tif]

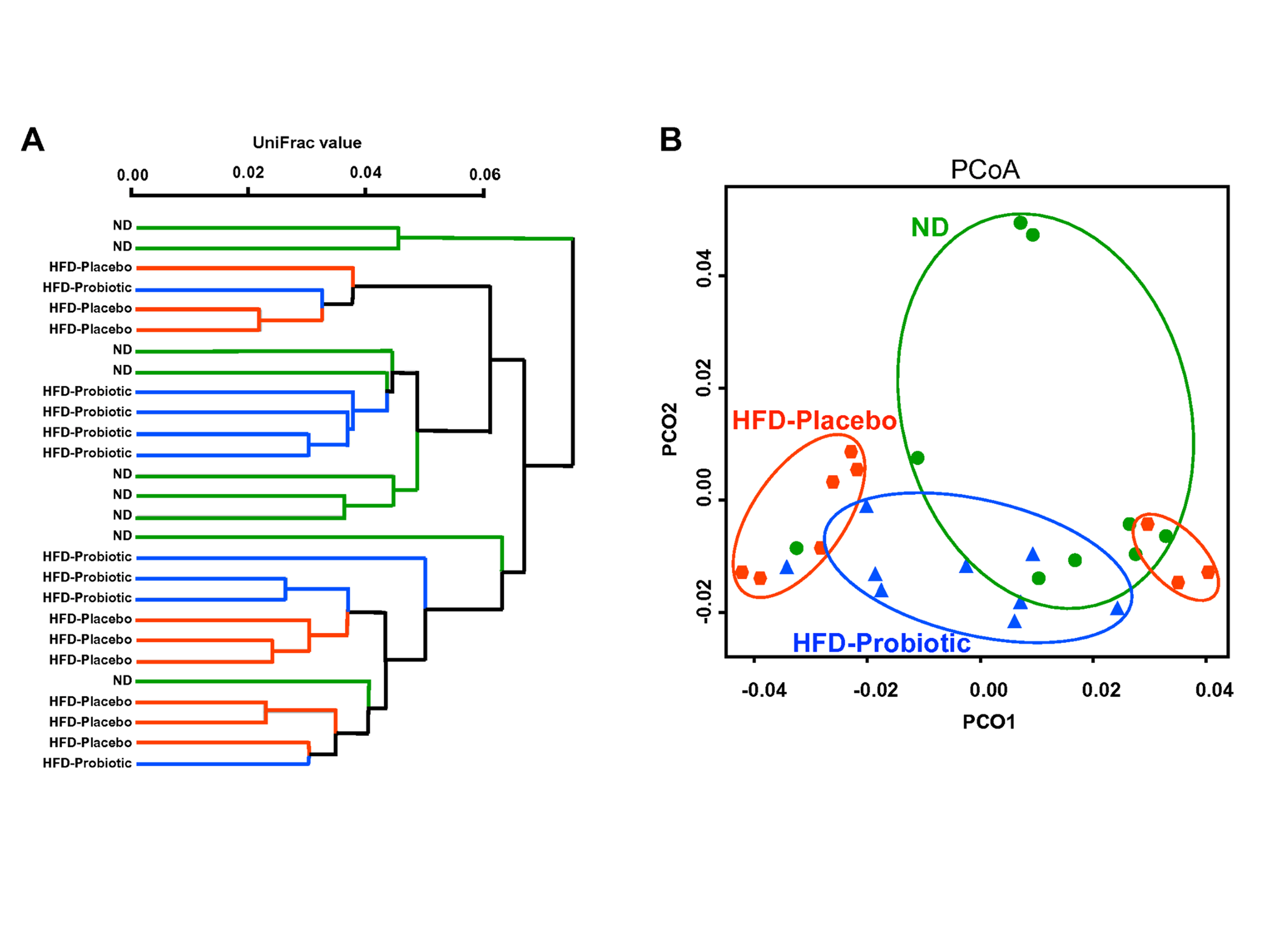

Supplement: Figure S2 — Clustering of samples based on gut microbiota communities. (A) UPGMA (Unweighted Pair-Group Method with Arithmetic mean) of samples from ND, HFD+placebo and HFD+probiotic group. (B) PCoA (Principal Coordinates Analysis) of samples from ND, HFD+placebo and HFD+probiotic group. (TIF) [file pone.0059470.s002.tif]

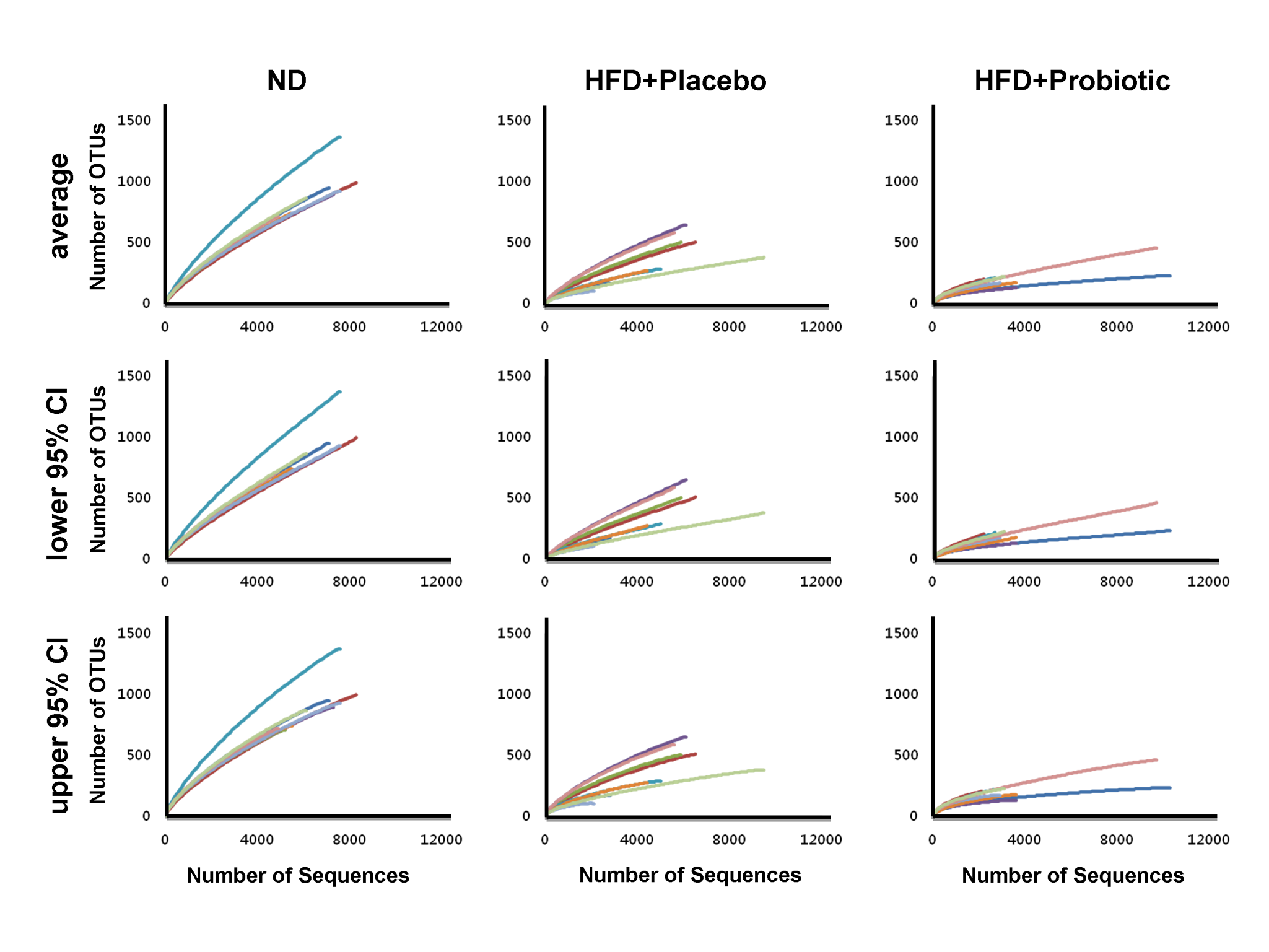

Supplement: Figure S3 — Rarefaction curves including 95% confidence intervals. Rarefaction analysis of 151,061 pyrosequencing reads of 16S rRNA from faeces at a 97 percent sequence similarity cut-off value. Rarefaction curves including 95% confidence intervals were constructed using MOTHUR software. (TIF) [file pone.0059470.s003.tif]
