# Supplementary material for: Supplementation of Lactobacillus curvatus HY7601 and Lactobacillus plantarum KY1032 in Diet-Induced Obese Mice Is Associated with Gut Microbial Changes and Reduction in Obesity
Source: PLoS One. 2013 Mar 21;8(3):e59470. doi: 10.1371/journal.pone.0059470 (PMC3605452; doi:10.1371/journal.pone.0059470)
Supplement: Table S1 — Composition of experimental diet. (DOC) [file pone.0059470.s004.doc]

**Table S1 Composition of experimental diet (unit: gram)**

| Ingredient (g) | ND | HFD |
| --- | --- | --- |
| Casein | 200.00 | 200.0 |
| D,L-methionine | 3.00 | 3.00 |
| Corn starch | 150.00 | 111.00 |
| Sucrose | 500.00 | 370.00 |
| Cellulose powder | 50.00 | 50.00 |
| Corn oil | 50.00 | 30.00 |
| Lard | — | 170.00 |
| Mineral mixture^a^ | 35.00 | 42.00 |
| Vitamin mixture^b^ | 10.00 | 12.00 |
| Choline bitartrate | 2.00 | 2.00 |
| Cholesterol | — | 10.00 |
| tert-Butylhydroquinone | 0.01 | 0.04 |
| Total (g) | 1000.0 | 1000.0 |

ND, normal diet control group; HFD, high-fat diet control group;

^a^ AIN-76 mineral mixture contained in (g/kg of mixture): calcium phosphate dibasic, 500.0; sodium chloride, 74.00; potassium citrate H_2_O, 222.00; potassium sulfate, 52.00; magnesium oxide, 24.00; manganous carbonate, 3.50; ferric citrate U.S.P, 6.00; zinc carbonate, 1.60; cupric carbonate, 0.30; potassium lodate, 0.01; sodium selenite, 0.01; chromium potassium sulfate 12H_2_O, 0.55; sucrose, finely powdered, 118.03.

^b^ AIN-76 vitamin mixture contained in (g/kg of mixture): thiamine HCl, 0.60; riboflavin, 0.60; pyridoxine HCl, 0.70; niacin, 3.00; calcium pantothenate, 1.60; folic acid, 0.20; biotin, 0.02; vitamin B12 (0.1%), 1.00; vitamin A palmitate (500,000 IU/g), 0.80; vitatmin D3 (400,000 IU/g), 0.25; vitamin E acetate (500 IU/g); 10.00; menadione sodium bisulfate, 0.08; sucrose, finely powdered, 981.15.
