# Supplementary material for: Supplementation of Lactobacillus curvatus HY7601 and Lactobacillus plantarum KY1032 in Diet-Induced Obese Mice Is Associated with Gut Microbial Changes and Reduction in Obesity
Source: PLoS One. 2013 Mar 21;8(3):e59470. doi: 10.1371/journal.pone.0059470 (PMC3605452; doi:10.1371/journal.pone.0059470)
Supplement: Table S2 — Catalog numbers of Taqman probes. (DOC) [file pone.0059470.s005.doc]

**Table S2** Catalog numbers of Taqman probes

| Taqman probe | Catalog number |
| --- | --- |
| Glyceraldehyde-3-phosphate dehydrogenase (GAPDH) | Mm99999915_g1 |
| Tumor necrosis factor-α (TNF-α) | Mm00443258_m1 |
| Interleukin-6 (IL-6) | Mm00446190_m1 |
| Interleukin-1β (IL-1β) | Mm01336189_m1 |
| Monocyte chemotactic protein-1 (MCP-1) | Mm00441242_m1 |
| Uncoupling protein-1 (UCP1) | Mm01244861_m1 |
| Uncoupling protein-2 (UCP2) | Mm00627597_m1 |
| PPARγ coactivator-1α (PGC-1α) | Mm01208835_m1 |
| Carnitine palmitoyl transferase-1 (CPT-1) | Mm00550438_m1 |
| Carnitine palmitoyl transferase 2 (CPT-2) | Mm00487205_m1 |
| Acyl-CoA oxidase-1 (ACOX-1) | Mm00443579_m1 |
| Peroxisome proliferative activated receptor-α (PPARα) | Mm00440939_m1 |
| Peroxisome proliferative activated receptor-γ (PPARγ) | Mm00440945_m1 |
| Sterol regulatory element binding protein-1 (SREBP-1) | Mm01138344_m1 |
| Fatty acid synthase (FAS) | Mm00662319_m1 |
| Stearoyl-CoA desaturase-1 (SCD1) | Mm00772290_m1 |
| Lipoprotein lipase (LPL) | Mm00434770_m1 |
| Hormone sensitive lipase (HSL) | Mm00495359_m1 |
| Sterol regulatory element binding protein-2 (SREBP-2) | Mm01306294_m1 |
| HMG-CoA reductase (HMGCR) | Mm01282499_m1 |
| Cholesterol 7 alpha-hydroxylase (CYP7A1) | Mm00484152_m1 |
| Low density lipoprotein receptor (LDLR) | Mm00440169_m1 |
