# Supplementary material for: Supplementation of Lactobacillus curvatus HY7601 and Lactobacillus plantarum KY1032 in Diet-Induced Obese Mice Is Associated with Gut Microbial Changes and Reduction in Obesity
Source: PLoS One. 2013 Mar 21;8(3):e59470. doi: 10.1371/journal.pone.0059470 (PMC3605452; doi:10.1371/journal.pone.0059470)
Supplement: Table S3 — The number of sequences analyzed, observed OTUs and estimated OTUs. (DOC) [file pone.0059470.s006.doc]

**Table S3 The number of sequences analyzed, observed OTUs and estimated OTUs**

|  | ND | HFD-placebo | HFD-probiotic |
| --- | --- | --- | --- |
| Mean reads | 6564±406 | 5274±730 | 4464±1055 |
| Mean OTUs |  |  |  |
| Observed | 893±64 | 402±67^†††^ | 219±36^*^ |
| ACE estimation | 1940±159 | 766±151^†††^ | 368±85^*^ |

Data shown as the means ± SE. Significant differences between groups were determined using unpaired Student’s t-test. Significant differences between HFD versus ND are indicated as ^†††^p<0.001. Significant differences between HFD+probiotic versus HFD+placebo are indicated as ^*^p<0.05.
