# Supplementary material for: Supplementation of Lactobacillus curvatus HY7601 and Lactobacillus plantarum KY1032 in Diet-Induced Obese Mice Is Associated with Gut Microbial Changes and Reduction in Obesity
Source: PLoS One. 2013 Mar 21;8(3):e59470. doi: 10.1371/journal.pone.0059470 (PMC3605452; doi:10.1371/journal.pone.0059470)
Supplement: Table S5 — The relative abundance at the phylum level. (DOC) [file pone.0059470.s008.doc]

**Table S5 The relative abundance at the phylum level**

|  | ND | HFD-placebo | HFD-probiotic |
| --- | --- | --- | --- |
| *Firmicutes* | 53.923±4.040 | 64.570±6.946 | 62.652±4.082 |
| *Bacteroidetes* | 36.074±4.900 | 30.756±7.012 | 36.349±3.988 |
| *Tenericutes* | 2.435±1.961 | 0.024±0.012^†††^ | 0.812±0.481 |
| *Verrucomicrobia* | 6.902±4.62 | 4.333±1.767 | 0^***^ |
| *Proteobacteria* | 0.392±0.133 | 0.201±0.048 | 0.051±0.020^*^ |

Data shown as the means ± SE. Values presented are percentage of relative abundance with respect to total bacterial sequences. Significant differences between groups using Kruskal–Wallis one-way analysis of variance with Bonferroni correction. Significant differences between HFD versus ND are indicated as ^†††^p<0.001. Significant differences between HFD+probiotic versus HFD+placebo are indicated as ^*^p<0.05, ^***^p<0.001.
