# Supplementary material for: Supplementation of Lactobacillus curvatus HY7601 and Lactobacillus plantarum KY1032 in Diet-Induced Obese Mice Is Associated with Gut Microbial Changes and Reduction in Obesity
Source: PLoS One. 2013 Mar 21;8(3):e59470. doi: 10.1371/journal.pone.0059470 (PMC3605452; doi:10.1371/journal.pone.0059470)
Supplement: Table S6 — Microbial species elevated by diet-induced obesity. (DOC) [file pone.0059470.s009.doc]

**Table S6 Microbial species elevated by diet-induced obesity**

| Phylum | Species | ND | HFD-placebo | HFD-probiotic |
| --- | --- | --- | --- | --- |
| *Firmicutes* | 4P000374_s | 0.896±0.501 | 4.084±0.915^†^ | 5.754±2.011 |
| *Firmicutes* | 4P001160_s | 0.043±0.011 | 0.354±0.055^†††^ | 0.656±0.103 |
| *Firmicutes* | EF098132_s | 1.549±0.886 | 15.802±3.120^††^ | 11.134±2.423 |
| *Firmicutes* | EU511797_s | 0.873±0.505 | 9.568±2.008^††^ | 5.617±1.156 |
| *Firmicutes* | EU509104_g_uc | 0.010±0.008 | 0.185±0.058^††^ | 0.203±0.054 |
| *Firmicutes* | *Clostridium cocleatum* | 0.094±0.075 | 0.692±0.162^††^ | 0.385±0.081 |
| *Firmicutes* | *Clostridium*_g6_uc | 0.001±0.001 | 0.037±0.013^†^ | 0.010±0.006 |

The relative abundance of 7 species was significantly elevated by diet-induced obesity. Data shown as the means ± SE. Values presented are percentage of relative abundance with respect to total bacterial sequences. Significant differences between HFD versus ND are indicated as ^†^p<0.05, ^††^p<0.01, ^†††^p<0.001. uc; unclassifed
