# Supplementary material for: Supplementation of Lactobacillus curvatus HY7601 and Lactobacillus plantarum KY1032 in Diet-Induced Obese Mice Is Associated with Gut Microbial Changes and Reduction in Obesity
Source: PLoS One. 2013 Mar 21;8(3):e59470. doi: 10.1371/journal.pone.0059470 (PMC3605452; doi:10.1371/journal.pone.0059470)
Supplement: Table S7 — Microbial species reduced by diet-induced obesity. (DOC) [file pone.0059470.s010.doc]

**Table S7 Microbial species reduced by diet-induced obesity**

| Phylum | Species | ND | HFD-placebo | HFD-probiotic |
| --- | --- | --- | --- | --- |
| *Firmicutes* | *Firmicutes*_uc_s | 0.035±0.007 | 0.002±0.002^††^ | 0 |
| *Firmicutes* | *Clostridia*_uc_s | 0.746±0.083 | 0.131±0.052^††^ | 0.085±0.046 |
| *Firmicutes* | *Clostridiales*_uc_s | 7.381±0.896 | 3.026±0.475^†††^ | 1.745±0.264 |
| *Firmicutes* | AB034128_f_uc_s | 0.019±0.006 | 0^†^ | 0 |
| *Firmicutes* | EF686514_f_uc_s | 1.431±0.324 | 0^†††^ | 0.080±0.039^*^ |
| *Firmicutes* | EF604607_s | 0.194±0.145 | 0^†^ | 0 |
| *Firmicutes* | EF604608_f_uc_s | 0.819±0.197 | 0.002±0.002^†††^ | 0.031±0.017 |
| *Firmicutes* | EF406702_f_uc_s | 0.083±0.036 | 0.003±0.003^††^ | 0 |
| *Firmicutes* | GU112196_f_uc_s | 0.011±0.005 | 0^†^ | 0.007±0.007 |
| *Firmicutes* | EU508511_s | 0.486±0.200 | 0^††^ | 0.480±0.178^*^ |
| *Firmicutes* | 4P001304_s | 0.133±0.047 | 0.016±0.016^†^ | 0.296±0.077^**^ |
| *Firmicutes* | EF404684_g_uc | 0.587±0.303 | 0.031±0.017^††^ | 0.038±0.020 |
| *Firmicutes* | EU453981_s | 0.015±0.004 | 0^†^ | 0.045±0.013^**^ |
| *Firmicutes* | *Clostridium*_g12_uc | 0.193±0.034 | 0.080±0.021^†^ | 0.078±0.020 |
| *Firmicutes* | DQ808472_g_uc | 0.042±0.012 | 0.006±0.004^†^ | 0 |
| *Firmicutes* | EF406618_g_uc | 0.149±0.029 | 0.019±0.007^††^ | 0.031±0.020 |
| *Firmicutes* | EF406503_s | 3.678±0.854 | 0.002±0.002^†††^ | 0.015±0.015 |
| *Firmicutes* | EF406745_g_uc | 0.532±0.091 | 0.002±0.002^†††^ | 0 |
| *Firmicutes* | EF406647_s | 0.087±0.016 | 0^†††^ | 0 |
| *Firmicutes* | EF603632_g_uc | 0.515±0.085 | 0.102±0.042^††^ | 0.067±0.057 |
| *Firmicutes* | EU454963_g_uc | 0.564±0.166 | 0.018±0.006^††^ | 0.037±0.015 |
| *Firmicutes* | 4P001325_g_uc | 0.153±0.055 | 0.064±0.060^†^ | 0.032±0.020 |
| *Firmicutes* | EU453505_g_uc | 0.058±0.024 | 0.002±0.002^††^ | 0.011±0.008 |
| *Firmicutes* | EU509117_s | 0.121±0.030 | 0.026±0.013^†^ | 0.103±0.063 |
| *Firmicutes* | *Roseburia*_uc | 0.036±0.009 | 0.007±0.004^†^ | 0.019±0.006 |
| *Firmicutes* | *Erysipelotrichales*_uc_s | 0.580±0.148 | 0.034±0.011^††^ | 0.240±0.158 |
| *Firmicutes* | *Erysipelotrichi*_uc_s | 0.861±0.185 | 0.036±0.016^††^ | 0.442±0.223 |
| *Bacteroidetes* | *Bacteroidia*_uc_s | 0.322±0.072 | 0.073±0.028^†^ | 0.019±0.011 |
| *Bacteroidetes* | *Bacteroidales*_uc_s | 0.858±0.173 | 0.192±0.078^†^ | 0.049±0.015 |
| *Bacteroidetes* | EF406830_g_uc | 1.387±0.342 | 0.208±0.133^†^ | 0.263±0.054 |
| *Tenericutes* | EF406813_s | 1.910±1.860 | 0^†^ | 0.021±0.014 |
| *Tenericutes* | EF445272_f_uc_s | 0.064±0.025 | 0^†^ | 0.027±0.027 |
| *Tenericutes* | EU381813_f_uc_s | 0.022±0.009 | 0^†^ | 0.011±0.006 |

The relative abundance of 33 species was significantly reduced by diet-induced obesity, among which 4 species increased in HFD-probiotic group. Data shown as the means ± SE. Values presented are percentage of relative abundance with respect to total bacterial sequences. Significant differences between HFD versus ND are indicated as ^†^p<0.05, ^††^p<0.01, ^†††^p<0.001. Significant differences between HFD+probiotic versus HFD+placebo are indicated as ^*^p<0.05, ^**^p<0.01. uc; unclassified
