# Supplementary material for: Supplementation of Lactobacillus curvatus HY7601 and Lactobacillus plantarum KY1032 in Diet-Induced Obese Mice Is Associated with Gut Microbial Changes and Reduction in Obesity
Source: PLoS One. 2013 Mar 21;8(3):e59470. doi: 10.1371/journal.pone.0059470 (PMC3605452; doi:10.1371/journal.pone.0059470)
Supplement: Table S8 — Microbial species elevated in mice receiving probiotic treatment. (DOC) [file pone.0059470.s011.doc]

**Table S8 Microbial species elevated by** probiotic treatment

| Phylum | Species | ND | HFD-placebo | HFD-probiotic |
| --- | --- | --- | --- | --- |
| *Firmicutes* | *Lactobacillus curvatus* | 0 | 0 | 0.362±0.070^***^ |
| *Firmicutes* | *Lactobacillus plantarum* | 0 | 0 | 0.065±0.021^**^ |
| *Firmicutes* | EF604612_s | 0.282±0.087 | 0.213±0.058 | 1.028±0.218^**^ |
| *Firmicutes* | EF406869_s | 0.020±0.014 | 0.008±0.004 | 0.111±0.051^*^ |
| *Firmicutes* | DQ015059_s | 0.093±0.024 | 0.192±0.042 | 0.459±0.071^*^ |
| *Firmicutes* | EU508225_g_uc | 0.219±0.089 | 0.109±0.028 | 0.374±0.093^*^ |
| *Firmicutes* | EU508225_s | 0.266±0.085 | 0.265±0.082 | 0.970±0.231^*^ |
| *Firmicutes* | EF406849_g_uc | 0.104±0.023 | 0.131±0.022 | 0.263±0.052^*^ |
| *Firmicutes* | EU508133_s | 0.011±0.005 | 0.025±0.013 | 0.218±0.089^*^ |
| *Firmicutes* | EF604613_s | 0.209±0.059 | 0.103±0.022 | 0.528±0.115^*^ |
| *Actinobacteria* | *Bifidobacterium pseudolongum* | 0.036±0.012 | 0.009±0.006 | 0.092±0.023^**^ |

The relative abundance of 11 species not associated with changes caused by diet-induced obesity was significantly elevated in mice receiving probiotic treatment. Data shown as the means ± SE. Values presented are percentage of relative abundance with respect to total bacterial sequences. Significant differences between HFD+probiotic versus HFD+placebo are indicated as ^*^p<0.05, ^**^p<0.01, ^***^p<0.001. uc; unclassifed
