# Supplementary material for: Supplementation of Lactobacillus curvatus HY7601 and Lactobacillus plantarum KY1032 in Diet-Induced Obese Mice Is Associated with Gut Microbial Changes and Reduction in Obesity
Source: PLoS One. 2013 Mar 21;8(3):e59470. doi: 10.1371/journal.pone.0059470 (PMC3605452; doi:10.1371/journal.pone.0059470)
Supplement: Table S9 — Microbial species reduced in mice receiving probiotic treatment. (DOC) [file pone.0059470.s012.doc]

**Table S9 Microbial species reduced by probiotic treatment**

| Phylum | Species | ND | HFD-placebo | HFD-probiotic |
| --- | --- | --- | --- | --- |
| *Firmicutes* | EF098132_g_uc | 0.638±0.216 | 0.841±0.230 | 0.157±0.048^*^ |
| *Firmicutes* | EU511797_g_uc | 0.441±0.143 | 0.565±0.158 | 0.095±0.040^*^ |
| *Firmicutes* | *Clostridiaceae*_uc_s | 0.090±0.021 | 0.029±0.017 | 0^*^ |
| *Firmicutes* | EF603943_g_uc_uc_s | 0.088±0.049 | 0.038±0.015 | 0^*^ |
| *Firmicutes* | AJ400262_s | 0.087±0.040 | 0.101±0.023 | 0.022±0.014^*^ |
| *Verrucomicrobia* | *Akkermansia muciniphila* | 6.253±4.241 | 4.137±1.697 | 0^*^ |
| *Proteobacteria* | *Escherichia coli* group | 0.130±0.087 | 0.044±0.029 | 0^*^ |

The relative abundance of 7 species not associated with changes caused by diet-induced obesity was significantly reduced in mice receiving probiotic treatment. Data shown as the means ± SE. Values presented are percentage of relative abundance with respect to total bacterial sequences. Significant differences between HFD+probiotic versus HFD+placebo are indicated as ^*^p<0.05. uc; unclassified
