# Supplementary material for: Supplementation of Lactobacillus curvatus HY7601 and Lactobacillus plantarum KY1032 in Diet-Induced Obese Mice Is Associated with Gut Microbial Changes and Reduction in Obesity
Source: PLoS One. 2013 Mar 21;8(3):e59470. doi: 10.1371/journal.pone.0059470 (PMC3605452; doi:10.1371/journal.pone.0059470)
Supplement: Table S10 — Comparison between 16 s RNA gene sequenced using universal primers and pyrosequencing primers. (DOCX) [file pone.0059470.s013.docx]

**Table S10 Comparison between 16s RNA gene sequenced using universal primers and pyrosequencing primers.**

|  | Sequences |
| --- | --- |
| *Lactobacillus curvatus* HY7601 16s RNA gene sequenced using universal primers (27F, 1492R) | ATACATGCAAGTCGAACGCACTCTCGTTAGATTGAAGAAGCTTGCTTCTGATTGATAACATTTGAGTGAGTGGCGGACGGGTGAGTAACACGTGGGTAACCTGCCCTAAAGTGGGGGATAACATTTGGAAACAGATGCTAATACCGCATAAAACCTAGCACCGCATGGTGCAAGGTTGAAAGATGGTTTCGGCTATCACTTTAGGATGGACCCGCGGTGCATTAGTTAGTTGGTGAGGTAAAGGCTCACCAAGACCGTGATGCATAGCCGACCTGAGAGGGTAATCGGCCACACTGGGACTGAGACACGGCCCAGACTCCTACGGGAGGCAGCAGTAGGGAATCTTCCACAATGGACGAAAGTCTGATGGAGCAACGCCGCGTGAGTGAAGAAGGTTTTCGGATCGTAAAACTCTGTTGTTGGAGAAGAACGTATTTGATAGTAACTGATCAGGTAGTGACGGTATCCAACCAGAAAGCCACGGCTAACTACGTGCCAGCAGCCGCGGTAATACGTAGGTGGCAAGCGTTGTCCGGATTTATTGGGCGTAAAGCGAGCGCAGGCGGTTTCTTAAGTCTGATGTGAAAGCCTTCGGCTCAACCGAAGAAGTGCATCGGAAACTGGGAAACTTGAGTGCAGAAGAGGACAGTGGAACTCCATGTGTAGCGGTGAAATGCGTAGATATATGGAAGAACACCAGTGGCGAAGGCGGCTGTCTGGTCTGTAACTGACGCTGAGGCTCGAAAGCATGGGTAGCAAACAGGATTAGATACCCTGGTAGTCCATGCCGTAAACGATGAGTGCTAGGTGTTGGAGGGTTTCCGCCCTTCAGTGCCGCAGCTAACGCATTAAGCACTCCGCCTGGGGAGTACGACCGCAAGGTTGAAACTCAAAGGAATTGACGGGGGCCCGCACAAGCGGTGGAGCATGTGGTTTAATTCGAAGCAACGCGAAGAACCTTACCAGGTCTTGACATCCTTTGACCACTCTAGAGATAGAGCTTTCCCTTCGGGGACAAAGTGACAGGTGGTGCATGGTTGTCGTCAGCTCGTGTCGTGAGATGTTGGGTTAAGTCCCGCAACGAGCGCAACCCTTATTACTAGTTGCCAGCATTTAGTTGGGCACTCTAGTGAGACTGCCGGTGACAAACCGGAGGAAGGTGGGGACGACGTCAAATCATCATGCCCCTTATGACCTGGGCTACACACGTGCTACAATGGATGGTACAACGAGTCGCGAGACCGCGAGGTTTAGCTAATCTCTTAAAACCATTCTCAGTTCGGATTGTAGGCTGCAACTCGCCTACATGAAGCCGGAATCGCTAGTAATCGCGGATCAGCATGCCGCGGTGAATACGTTCCCGGGCCTTGTACACACCGCCCGTCACACCATGAGAGTTTGTAACACCCAAAGCCGGTGAGGTAACCTTCGGGAGCCAGCCGTCTAAG |
| The representative *Lactobacillus curvatus* HY7601 16s RNA gene sequenced using pyrosequencing primers (9F, 541R) | CTAATACATGCAAGTCGAACGCACTCTCGTTAGATTGAAGAAGCTTGCTTCTGATTGATAACATTTGAGTGAGTGGCGGACGGGTGAGTAACACGTGGGTAACCTGCCCTAAAGTGGGGGATAACATTTGGAAACAGATGCTAATACCGCATAAAACCTAGCACCGCATGGTGCAAGGTTGAAAGATGGTTTCGGCTATCACTTTAGGATGGACCCGCGGTGCATTAGTTAGTTGGTGAGGTAAAGGCTCACCAAGACCGTGATGCATAGCCGACCTGAGAGGGTAATCGGCCACACTGGGACTGAGACACGGCCCAGACTCCTACGGGAGGCAGCAGTAGGGAATCTTCCACAATGGACGAAAGTCTGATGGAGCAACGCCGCGTGAGTGAAGAAGGTTTTCGGATCGTAAAACTCTGTTGTTGGAGAAGAACGTATTTGATAGTAACTGATCAGGTAGTGACGGTATCCAACCAGAAAGCCACGGCTAACT |
| *Lactobacillus plantarum* KY1032 16s RNA gene sequenced using universal primers (27F, 1492R) | CGAACTCTGGTATTGATTGGTGCTTGCATCATGATTTACATTTGAGTGAGTGGCGAACTGGTGAGTAACACGTGGGAAACCTGCCCAGAAGCGGGGGATAACACCTGGAAACAGATGCTAATACCGCATAACAACTTGGACCGCATGGTCCGAGCTTGAAAGATGGCTTCGGCTATCACTTTTGGATGGTCCCGCGGCGTATTAGCTAGATGGTGGGGTAACGGCTCACCATGGCAATGATACGTAGCCGACCTGAGAGGGTAATCGGCCACATTGGGACTGAGACACGGCCCAAACTCCTACGGGAGGCAGCAGTAGGGAATCTTCCACAATGGACGAAAGTCTGATGGAGCAACGCCGCGTGAGTGAAGAAGGGTTTCGGCTCGTAAAACTCTGTTGTTAAAGAAGAACATATCTGAGAGTAACTGTTCAGGTATTGACGGTATTTAACCAGAAAGCCACGGCTAACTACGTGCCAGCAGCCGCGGTAATACGTAGGTGGCAAGCGTTGTCCGGATTTATTGGGCGTAAAGCGAGCGCAGGCGGTTTTTTAAGTCTGATGTGAAAGCCTTCGGCTCAACCGAAGAAGTGCATCGGAAACTGGGAAACTTGAGTGCAGAAGAGGACAGTGGAACTCCATGTGTAGCGGTGAAATGCGTAGATATATGGAAGAACACCAGTGGCGAAGGCGGCTGTCTGGTCTGTAACTGACGCTGAGGCTCGAAAGTATGGGTAGCAAACAGGATTAGATACCCTGGTAGTCCATACCGTAAACGATGAATGCTAAGTGTTGGAGGGTTTCCGCCCTTCAGTGCTGCAGCTAACGCATTAAGCATTCCGCCTGGGGAGTACGGCCGCAAGGCTGAAACTCAAAGGAATTGACGGGGGCCCGCACAAGCGGTGGAGCATGTGGTTTAATTCGAAGCTACGCGAAGAACCTTACCAGGTCTTGACATACTATGCAAATCTAAGAGATTAGACGTTCCCTTCGGGGACATGGATACAGGTGGTGCATGGTTGTCGTCAGCTCGTGTCGTGAGATGTTGGGTTAAGTCCCGCAACGAGCGCAACCCTTATTATCAGTTGCCAGCATTAAGTTGGGCACTCTGGTGAGACTGCCGGTGACAAACCGGAGGAAGGTGGGGATGACGTCAAATCATCATGCCCCTTATGACCTGGGCTACACACGTGCTACAATGGATGGTACAACGAGTTGCGAACTCGCGAGAGTAAGCTAATCTCTTAAAGCCATTCTCAGTTCGGATTGTAGGCTGCAACTCGCCTACATGAAGTCGGAATCGCTAGTAATCGCGGATCAGCATGCCGCGGTGAATACGTTCCCGGGCCTTGTACACACCGCCCGTCACACCATGAGAGTTTGTAACACCCAAAGTCGGTGGGGTAACCTTTAGGAACCAGCCGCTAA |
| The representative *Lactobacillus plantarum* KY1032 16s RNA gene sequenced using pyrosequencing primers (9F, 541R) | CTAATACATGCAAGTCGAACGAACTCTGGTATTGATTGGTGCTTGCATCATGATTTACATTTGAGTGAGTGGCGAACTGGTGAGTAACACGTGGGAAACCTGCCCAGAAGCGGGGGATAACACCTGGAAACAGATGCTAATACCGCATAACAACTTGGACCGCATGGTCCGAGCTTGAAAGATGGCTTCGGCTATCACTTTTGGATGGTCCCGCGGCGTATTAGCTAGATGGTGGGGTAACGGCTCACCATGGCAATGATACGTAGCCGACCTGAGAGGGTAATCGGCCACATTGGGACTGAGACACGGCCCAAACTCCTACGGGAGGCAGCAGTAGGGAATCTTCCACAATGGACGAAAGTCTGATGGAGCAACGCCGCGTGAGTGAAGAAGGGTTTCGGCTCGTAAAACTCTGTTGTTAAAGAAGAACATATCTGAGAGTA |
